# Pulsatilla Decoction and its bioactive component β-peltatin induce G2/M cell cycle arrest and apoptosis in pancreatic cancer

**DOI:** 10.1186/s13020-023-00774-0

**Published:** 2023-05-28

**Authors:** Rong Wu, Zhichao Xi, Mengfan Liu, Hangui Ren, Rongchen Dai, Xue Jiang, Wan Najbah Nik Nabil, Yalin Wang, Jiling Feng, Qiong Chai, Qihan Dong, Hongxi Xu

**Affiliations:** 1 School of Pharmacy, Shanghai University of Traditional Chinese Medicine, Shanghai, 201203 China; 2 Engineering Research Center of Shanghai Colleges for TCM New Drug Discovery, Shanghai, 201203 China; 3 Pharmaceutical Services Program, Ministry of Health, Petaling Jaya, Selangor 46200 Malaysia; 4 Chinese Medicine Anti-Cancer Evaluation Program, Greg Brown Laboratory, Central Clinical School and Charles Perkins Centre, Faculty of Medicine and Health, The University of Sydney, Sydney, NSW 2006 Australia; 5 Department of Endocrinology, Royal Prince Alfred Hospital, Sydney, NSW 2050 Australia

**Keywords:** Pulsatilla Decoction, Pulsatilla, β-peltatin, Pancreatic cancer, G2/M arrest, Apoptosis

## Abstract

**Background:**

Pancreatic cancer (PAC), a malignancy that is fatal and commonly diagnosed at a late stage. Despite considerable advancements in cancer treatment, the survival rate of PAC remains largely consistent for the past 60 years. The traditional Chinese medicine formula Pulsatilla Decoction (PD) has been clinically used to treat inflammatory diseases for millennia and recently as a supplementary anti-cancer treatment in China. However, the bioactive ingredients and mechanisms underlying its anti-cancer effect remains unclear.

**Methods:**

The composition and quality control of PD were verified through analysis by high performance liquid chromatography. Cell viability was determined using Cell Counting Kit-8 assay. The cell cycle distribution was analyzed through PI staining and flow cytometry analysis, while apoptotic cells were measured by double staining with Annexin V-FITC and PI. We used immunoblotting to examine protein expressions. The in vivo effects of β-peltatin and podophyllotoxin were evaluated on a subcutaneously-xenografted BxPC-3 cell nude mice model.

**Results:**

The current study demonstrated that PD markedly inhibited PAC cell proliferation and triggered their apoptosis. Four herbal PD formula was then disassembled into 15 combinations of herbal ingredients and a cytotoxicity assay showed that the *Pulsatillae chinensis* exerted the predominant anti-PAC effect. Further investigation indicated that β-peltatin was potently cytotoxic with IC_50_ of ~ 2 nM. β-peltatin initially arrested PAC cells at G2/M phase, followed by apoptosis induction. Animal study confirmed that β-peltatin significantly suppressed the growth of subcutaneously-implanted BxPC-3 cell xenografts. Importantly, compared to podophyllotoxin that is the parental isomer of β-peltatin but clinically obsoleted due to its severe toxicity, β-peltatin exhibited stronger anti-PAC effect and lower toxicity in mice.

**Conclusions:**

Our results demonstrate that *Pulsatillae chinensis* and particularly its bioactive ingredient β-peltatin suppress PAC by triggering cell cycle arrest at G2/M phase and apoptosis.

**Supplementary Information:**

The online version contains supplementary material available at 10.1186/s13020-023-00774-0.

## Background

Pancreatic cancer (PAC) is currently the third leading cause of cancer mortality globally [1]. Of all cancer types, the five-year survival rate for PAC is the lowest of merely 11% [1, 2]. Although combination treatments, such as chemo-radiotherapy or combinatorial chemo-targeted therapy, are administered after tumor removal surgery, about 76% of patients still suffer recurrence within 2 years [2, 3].

Traditional Chinese Medicine (TCM) is a unique resource for research and discovery of new drugs. However, a TCM formula usually consists of multiple herbs and each of the herb contains numerous compounds, making it difficult to conclude which herb or compound has the prominent effect. Nevertheless, identifying the most bioactive compound is clinically relevant. When an herbal drug can be prepared with fewer ingredients, it reduces the drug amount for encapsulation, eases its intake, and ultimately improves drug compliance. Therefore, disassembling a formula into its herbal constituent is one of the effective approaches in identifying the bioactive compounds within a TCM formula.

Pulsatilla Decoction (PD) originates from the classical Chinese medicine book “Shang Han Treatise”, and consists of four herbs i.e., *Pulsatillae chinensis* (Bai Tou Weng, BTW), *Coptidis chinensis* (Huang Lian, HL), *Phellodendri Chinensis Cortex* (Huang Bai, HB), and *Fraxini Cortex* (Qin Pi, QP) in the weight ratio of 2.5:2:2:1 [4]. Modern pharmacological research has demonstrated that PD alleviates various diseases, including diarrhea, inflammatory bowel disease, *Escherichia coli* infection, vulvovaginal candidiasis, and cancer [4–10]. The preclinical evidence on the anticancer properties of PD mainly focuses on colorectal and colon cancer, while research on other types of cancer remains limited [4, 5].

The primary compounds isolated from the main herb, BTW, are triterpenoid saponins, phytosterone and anthocyanins [11]. Studies have demonstrated that anemoside B4, the major triterpenoid saponin from BTW, along with its metabolite 23-hydroxybetulinic acid, impeded multidrug-resistance and potentiates chemotherapy efficacy in numerous cancer cell lines, such as leukemia, lung, liver and breast cancers [12]. Anemoside A3, another metabolite of anemoside B4 found in smaller quantities in BTW, has been shown in preclinical studies to suppress the growth, angiogenesis and metastasis of breast cancer [12–14]. HL and HB are rich in alkaloids such as berberine, palmatine and coptisine [11]. Extensive preclinical studies have demonstrated that berberine possesses anticancer properties by inhibiting the growth and metastasis of numerous cancers *via* multiple signaling pathways [15, 16]. Fraxetin, aesculin, and aeculetin are bioactive compounds found in QP [11, 17]. Fraxetin has been more broadly and preclinically studied for its anticancer properties in various cancers such as prostate, breast, liver, and colon [17]. Although compounds have been isolated from the herbal component of PD [7, 11, 18], the bioactive ingredients and mechanisms underlying their anti-PAC effect remain elusive. Therefore, the aim of the current study was to explore the effective herb, active compounds and underlying mechanism of PD in inhibiting PAC.

## Methods

### Materials

We sourced the Chinese herbal granules of BTW, HB, QP, HL, from Sichuan New Green Pharmaceutical Technology Co., LTD (Sichuan, PR China). They were solubilized in dimethyl sulfoxide (St. Louis, MO), and kept at -20 °C. Anemoside B_4_, Berberine hydrochloride, Phellodendrine chloride, Aesculetin, Esculin were obtained from Shanghai Yuanye Biological Co., LTD (Shanghai, PR China). β-peltatin and podophyllotoxin were acquired from TianZhi Biological Co., LTD (Shanghai, PR China). Gemcitabine HCl (GEM) was purchased from Selleck Co., LTD (Shanghai, PR China). We obtained Cell Counting Kit-8 (CCK-8) from Taosu Biological Co., LTD (Shanghai, PR China), while RPMI-1640 medium from Gibco Company (Invitrogen, Carlsbad, CA). Trypsin and propidium iodide (PI) were procured from Sigma Company (St. Louis, MO). Annexin V-FITC/PI apoptosis detection kit was obtained from Meilun Biological Co., LTD (Shanghai, PR China).

### Cell culture

We obtained MIA PaCa-2 and BxPC-3, human PAC cell lines, from American Type Culture Collection (Rockville, USA). We cultured the cells in complete RPMI-1640 medium supplemented with 10% fetal bovine serum (Invitrogen, Carlsbad, CA), 1 mM L-glutamine and antibiotics (100 μg/mL streptomycin and 100 U/mL penicillin). The cells were then cultured in a 5% CO_2_ incubator under standard conditions (37 °C).

### Cell viability assay

We first seeded PAC cells in 96-well plate at a density of 3 × 10^3^ cells/well and incubated for one day. Next, the cells were treated with indicated concentrations of test substances for 24, 48 or 72 h. Cell Counting Kit-8 (CCK-8) was employed to evaluate cell viability, with absorbance at 450 nm being measured by a multifunctional microplate reader (FLUOstar Omega, BMG Labtech, Germany). By applying software GraphPad Prism 5 (La Jolla, California, USA), we calculated the IC_50_ by analysing the dose–response curves.

### High performance liquid chromatography (HPLC)

Briefly, PD was analyzed using HPLC, and Waters® 2695 was utilized for HPLC method conditions of PD. We maintained the Agilent® C18 column at 30 °C. The mixture of solution A (acetonitrile) and solution B (0.1% orthophosphoric acid) was used as the mobile phase, flowing at a rate of 1 mL/min. The injection volume of each sample was kept at 10 µL, and the elution was monitored at 210 nm. A gradient elution was performed using the following conditions: 5–10% A (0–5 min), 10–15% A (5–10 min), 15–20% A (10–20 min), 20–30% A (20–30 min), 30–50% A (30–40 min), 50–90% A (40–50 min), 90–5% A (50–60 min). The PD solution and five pharmacopoeia reference solutions: esculin, aesculetin, phellodendrine hydrochloride, berberine hydrochloride and anemoside B_4_ dissolved in methanol. HPLC analysis confirmed the presence of those reference substances in PD by comparing the peaks retention times and ultraviolet absorption (Additional file 1: Fig. S1).

### Isolation of active fraction and compound by structural characterization

The active fractions were initially obtained through isolation, which was followed by the isolation of active compounds, as described earlier (submitted). Commercial BTW extracts (https://www.tianjiangus.com/) were dissolved in water. The suspension was loaded onto RPS-C18 column. Following identification of an anti-proliferative activity in 65% methanol eluate, the fraction was applied to RPS-C18 column chromatography and the active compound was identified in elutes with 90% acetonitrile water using UPLC-Q-TOF/MS. In the second round, original BTW herb (https://www.shstcm.com/) was used for large scale isolation. After mixing the concentrate BTW extract with AB-8 macroporous adsorption resin, the mixture was then loaded onto column for elusion. We concentrated the bioactive fraction under reduced pressure at 60 °C and then freeze-dried. The target compound was identified by using 1H- and 13C-NMR. Its molecular was determined to be C_22_H_22_O_8_ using high-resolution mass spectrometry (HRESIMS).

### Cell cycle distribution analysis

PAC cells were cultured in 6-well plates with a seeding density of 3 × 10^5^ cells/well and incubated for 24 h. Then the cells were treated with varying concentrations of test substances for 12, 24 and 48 h. After harvesting the cells, we then fixed them overnight in 70% ethanol in phosphate-buffered saline. The cells were stained with PI, and then analyzed by a flow cytometer (BD Biosciences, San Jose, CA) following previous protocol [19].

### Annexin V-FITC/PI apoptosis detection

Cellular apoptosis was detected by the double staining of Annexin V-FITC and PI following the guidelines of the manufacturer. PAC cells (1 × 10^5^/well) were seeded into 12-well plates and cultured for one day before being treated for 48 h with test substances. After washing with phosphate-buffered saline, we incubated the cells with FITC and PI for 15 min, then analyzed apoptotic cells using flow cytometry and FlowJo software (version VX) as previously described [20].

### Immunoblotting

We lysed the cell pellet using a mixture of RIPA lysis buffer and protease inhibitor on ice. The protein quantification was performed using bicinchoninic acid assay, followed by electrophoresis and transfer as previously described [21]. The antibodies (human specific) for immunoblotting namely cyclin B1 (12231), CDK1(9116), p-CDC25C (Ser216, 4901), p-Histone H3 (Ser10, 3377S), PARP (9542), caspase 3 (9662S), caspase 9 (9502) and Bcl-2 (15071S) were supplied by Cell signaling Technology Company (Danvers, MA, USA). While antibodies for anti-p-CDK1 (Tyr15, sc-136014) and CDC25C (sc-13138) were from Santa Cruz Biotechnology Company (Dallas, TX, USA), β-actin (66009-1) and GAPDH (60004-1) were purchased from Proteintech Company (Shanghai, PR China).

### Animal experiments

The animal experiments carried out in the present study were authorized by Animal Center of Shanghai University of Traditional Chinese Medicine Committee, and we followed the institutional guidelines in providing animal care (NO. PZSHUTCM210903019 and NO. PZSHUTCM211115024). Female BALB/c nude mice and BALB/c mice, both six weeks of age, were supplied from the Chinese Academy of Science (Shanghai, China), and housed for 14 days under pathogen-free conditions. For toxicity evaluation study of β-peltatin and podophyllotoxin, 20 BALB/c mice were randomly allocated to two treatment groups after fasted for 12–16 h. β-peltatin and podophyllotoxin were dissolved in saline, then injected 60 mg/kg subcutaneously into the mice. The changes of general behavior and death of animals were observed continuously for the first 4 h and then daily for the following 17 days. Body weight and survival rate were also recorded daily.

For the experimental treatment study, BxPC-3 cells were administered *via* subcutaneous injection into the right flank of each mouse at a concentration of 2 × 10^6^ cell/mL. Upon the tumor reaching a size of 100 mm^3^, mice were allocated to vehicle group (50% propylene glycol in saline), β-peltatin group (15 mg/kg) and podophyllotoxin group (15 mg/kg) which were administrated intraperitoneally for once weekly. Both β-peltatin and podophyllotoxin were dissolved in propylene glycol in saline. Tumor volume were measured on alternate days by using (length x width^2^) /2. When tumors reached 1.0 cm^3^, the mice were euthanized, and both tumors and major organs were excised for analysis.

### Immunohistochemistry

After euthanasia, the tumors were excised and promptly fixed in 10% neutral-buffered paraformaldehyde. Next, the samples were paraffin-embedded and were cut into 5 μm slices. Staining with hematoxylin and eosin (H&E), Ki-67 (1:1000; abcam, ab16667), cleaved caspase 3 (1:200, Huaan, ET1608-64) and p-Histone H3 (Ser10,1:800) were then performed on the specimens. DPX was used to mount the sections for histological analysis.

### Statistical analysis

Mean ± S.D. values are presented for at least 3 independent experiments. SPSS version 21.0 was utilized for data analysis, and student’s two-tailed *t*-test was used to assess the significance of the difference between two groups. To compare multiple groups, we performed ANOVA with Fisher’s LSD multiple comparison test. Statistical significance was determined at the probability values of *p < 0.05, **p < 0.01, ***p < 0.001 compared to control.

## Results

### PD markedly decreases PAC cells viability.

The cytotoxicity of 13 classical Chinese herbal formulae from Chinese medicine book “Shang Han Treatise” (A-M, listed in Additional file 1: Table S1) on PAC cells was evaluated using the CCK-8 assay. As shown in Additional file 1: Fig. S2, after 72 h exposure of PAC cells to the 13 formulae at concentration of 100 μg/mL, certain formulae such as PD (A), Yinchenhao Tang Decoction (C) and Wuzhuyu Decoction (H), showed prominent cytotoxicity. Among them, PD (A) demonstrated the strongest cytotoxicity on the two PAC cell lines, Gemcitabine HCl (GEM), a clinically used anti-PAC agent, was set as a positive control.

**Fig. 1:**
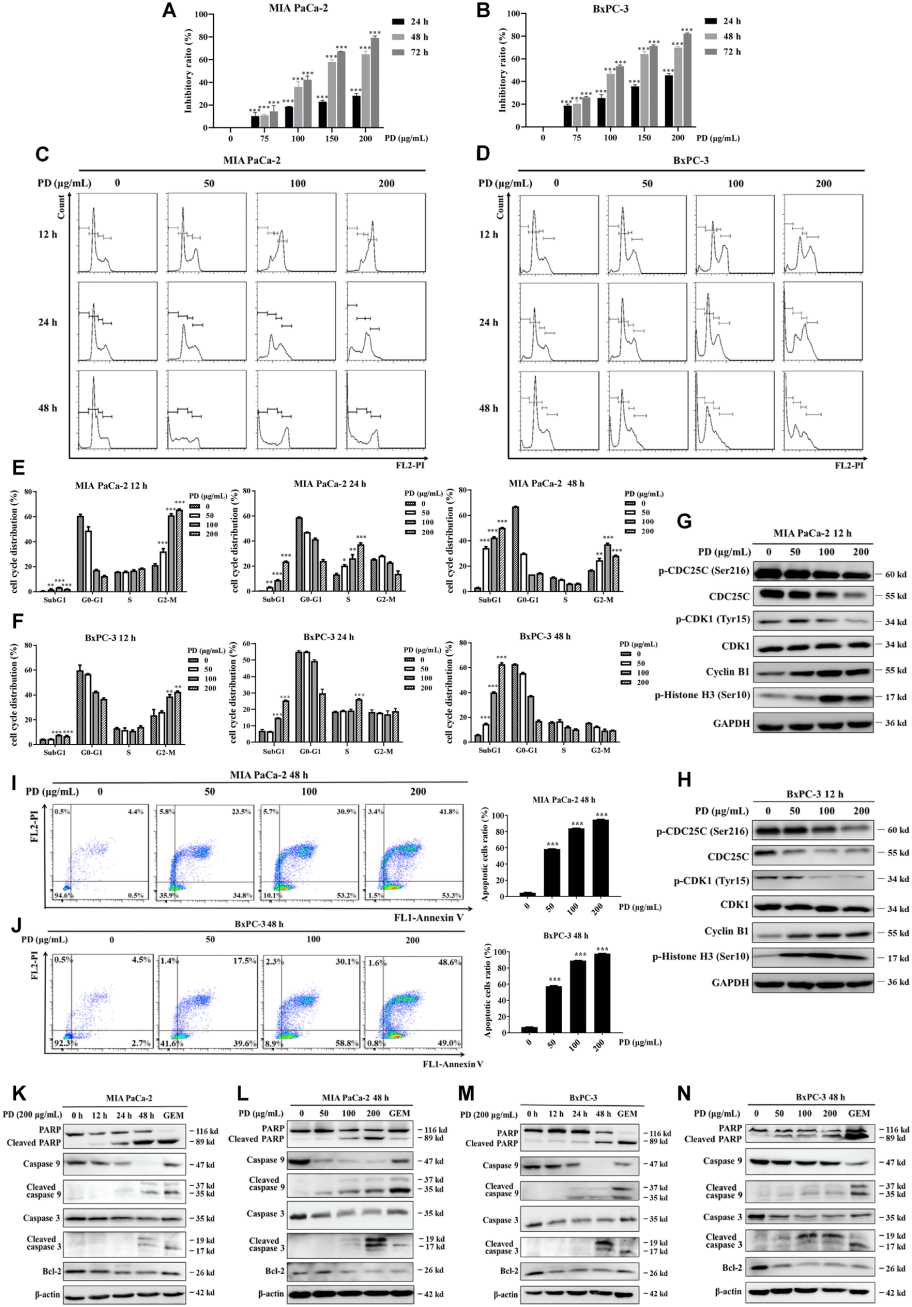
PD induces cell cycle arrest at G2/M phase and mitochondrial apoptosis in PAC cells. The viability of PAC cells (**A**, **B**) after PD treatment at concentrations of 0, 75, 100, 150 and 200 μg/mL was determined using CCK-8 for 24 h, 48 h and 72 h, respectively. Representative images of cell cycle distribution of PAC cells (**C**, **D**) after treated with PD (0–200 μg/mL) for 12–48 h, respectively. Quantification data of cell cycle distribution for PAC cells (**E**, **F**) are presented. The levels of G2/M phase-related regulatory proteins (p-CDC25C (Ser216), CDC25C, Cyclin B1, p-CDK1(Tyr15), CDK1 and p-Histone H3 (Ser10)) in PAC cells (**G**,** H**) were analyzed using immunoblotting following exposure to PD at concentrations of 0, 50, 100 and 200 μg/mL for 12 h. The expression levels were normalized to GAPDH. Double staining with Annexin V-FITC and PI was conducted to evaluate apoptosis in PAC cells (**I**, **J**) following PD treatment for 48 h (0–200 μg/mL) and then quantified (right panel). Apoptosis-associated protein expression was determined using immunoblotting in MIA PaCa-2 (**K**, **L**) and BxPC-3 (**M**, **N**) following PD treatment at concentrations of 0, 50, 100 and 200 μg/mL for duration of 12, 24 and 48 h. The expression levels were normalized to β-actin. 0.5 μM of GEM was chosen as the positive control. Results are presented as mean ± S.D. of triplicate independent experiments. *p < 0.05, **p < 0.01, ***p < 0.001 compared with the control

To examine the inhibitory action of PD on PAC cells, two PAC cell lines were exposed to PD at different concentrations for a period of 24 h, 48 h and 72 h. After a 48 h exposure to PD, the half-maximal inhibitory concentration (IC_50_) values were 130.61 μg/mL ± 27.74 for MIA PaCa-2 cells and 119.62 μg/mL ± 11.59 for BxPC-3 cells. Whereas IC_50_ for 72 h treatment were 128.92 μg/mL ± 15.25 and 99.84 μg/mL ± 16.36, respectively. These data showed that PD dose- and time-dependently decreased the viability of PAC cells (Fig. 1A, B).

### PD triggers PAC cells to arrest at G2/M phase and undergo apoptosis.

To investigate the effects of PD on cell cycle progression, PAC cells were treated with PD at different concentrations. After 12–48 h, the cells were then harvested and analyzed with PI-staining flow cytometry. Following 12 h exposure of 200 μg/mL PD, there was a notable increase in the number of cells in the G2/M phase, rising from 20% to 66.1% in MIA PaCa-2 cells and 20.4% to 42.3% in BxPC-3 cells (Fig. 1C–F). CDC25C phosphatase dephosphorylates CDK1, then activated cyclin B1-CDK1 complex drives cell progression through G2/M checkpoint and enter mitosis, accompanied with increased p-Histone H3 (Ser10) expression [22, 23]. After 12 h treatment of PD, the expression levels of G2/M phase-related proteins, such as p-CDC25C (Ser216), CDC25C and p-CDK1 (Tyr15), were decreased. Conversely, a dose-dependent upregulation of cyclin B1 and p-Histone H3(Ser10) protein levels was observed (Fig. 1G, H). These results suggested that PD arrested cell cycle at G2/M phase and possibly at M phase.

Notably, the accumulation of SubG1 phase cells were markedly enhanced following 24 and 48 h of PD treatment (Fig. 1C–F). Hence, we performed Annexin V/PI double staining assay to evaluate whether PD induced cell apoptosis. As shown in Fig. 1I and J, a dose-dependent induction of apoptosis was observed in PAC cells after 48 h of PD treatment, with increments were from 4.9% to 95.1%, and from 7.2% to 97.6%, respectively. The immunoblotting results verified that PD treatment resulted in a decrease in the expression of anti-apoptotic protein Bcl-2 and an increase in the expression of cleaved PARP, cleaved caspase 3, and cleaved caspase 9 in a dose- and time-dependent manner (Fig. 1K–N). Overall, the data suggest that PD triggers cell cycle arrest at G2/M phase and promotes apoptosis.

### BTW from PD exerts the predominant anti-PAC effect.

**Fig. 2:**
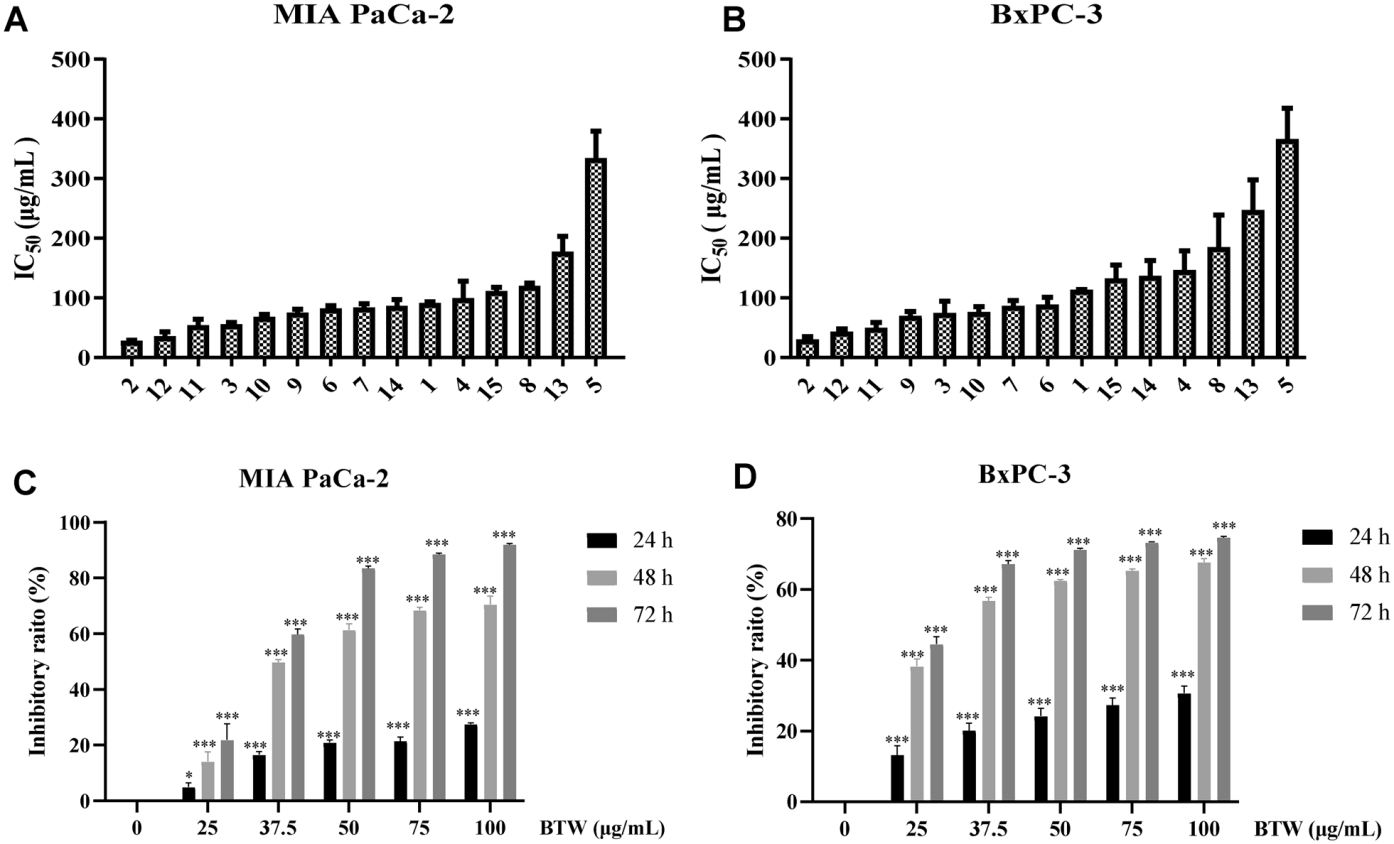
The cytotoxicity of 15 disassembled groups from PD on PAC cells. The IC_50_ values of 15 disassembled groups on PAC cells (**A**, **B**) at 72 h. The inhibitory effect on PAC cells (**C**, **D**) after treated with BTW at concentrations of 0, 25, 37.5, 50, 75 and 100 μg/mL for a period time of 24, 48 and 72 h. Data are presented as mean ± S.D. of triplicate independent experiments. *p < 0.05, ***p < 0.001 compared with the control

To identify which herb that predominates the inhibitory effect of PD on PAC cells, the herbal content of PD were proportionately separated according to their original weight ratio in PD (BTW:HL:HB:QP = 2.5:2:2:1). The separated herbal contents were then variedly combined producing 15 dissembled groups(Table 1). The cytotoxicity of 15 disassembled groups on PAC cells was evaluated using CCK-8 assay after 72 h. As shown in Fig. 2A, B**,** BTW (group 2) showed the strongest cytotoxicity against two cell lines, with IC_50_ value of 27.9 μg/mL ± 1.25 in MIA PaCa-2 cells and 30.24 μg/mL ± 4.45 in BxPC-3 cells, which was about fourfold lower than PD. Additionally, all the dissembled groups that contain BTW (group 6, 7, 9, 10, 11 and 12) showed greater cytotoxicity with lower IC_50_ value than PD, suggesting that BTW is the most effective ingredient contributing to the anti-pancreatic effect of PD. Therefore, CCK-8 assay was used to evaluate the inhibitory effect of BTW at different concentrations during a range of time periods in PAC cells (Fig. 2C, D). Our findings indicate that BTW exerts a dose- and time-dependent inhibitory effect on the growth of PAC cells.

**Table 1:** 15 disassembled groups from PD

Disassembled group	Herbal content
1	PD (BTW + HL + HB + QP)
2	BTW
3	HL
4	HB
5	QP
6	BTW + HB + QP
7	BTW + HL + QP
8	HB + HL + QP
9	BTW + HB + HL
10	BTW + HB
11	BTW + QP
12	BTW + HL
13	HB + QP
14	HB + HL
15	HL + QP

Herbal content of PD were proportionately separated according to their original weight ratio in PD (BTW:HL:HB:QP = 2.5:2:2:1), producing 15 dissembled groups. PD, Pulsatilla Decoction; BTW (Bai Tou Weng), *Pulsatillae chinensis*; HB (Huang Bai), *Phellodendron chinense*; HL (Huang Lian), *Coptis chinensis*; QP (Qin Pi), *Cortex fraxini*.

**Fig. 3:**
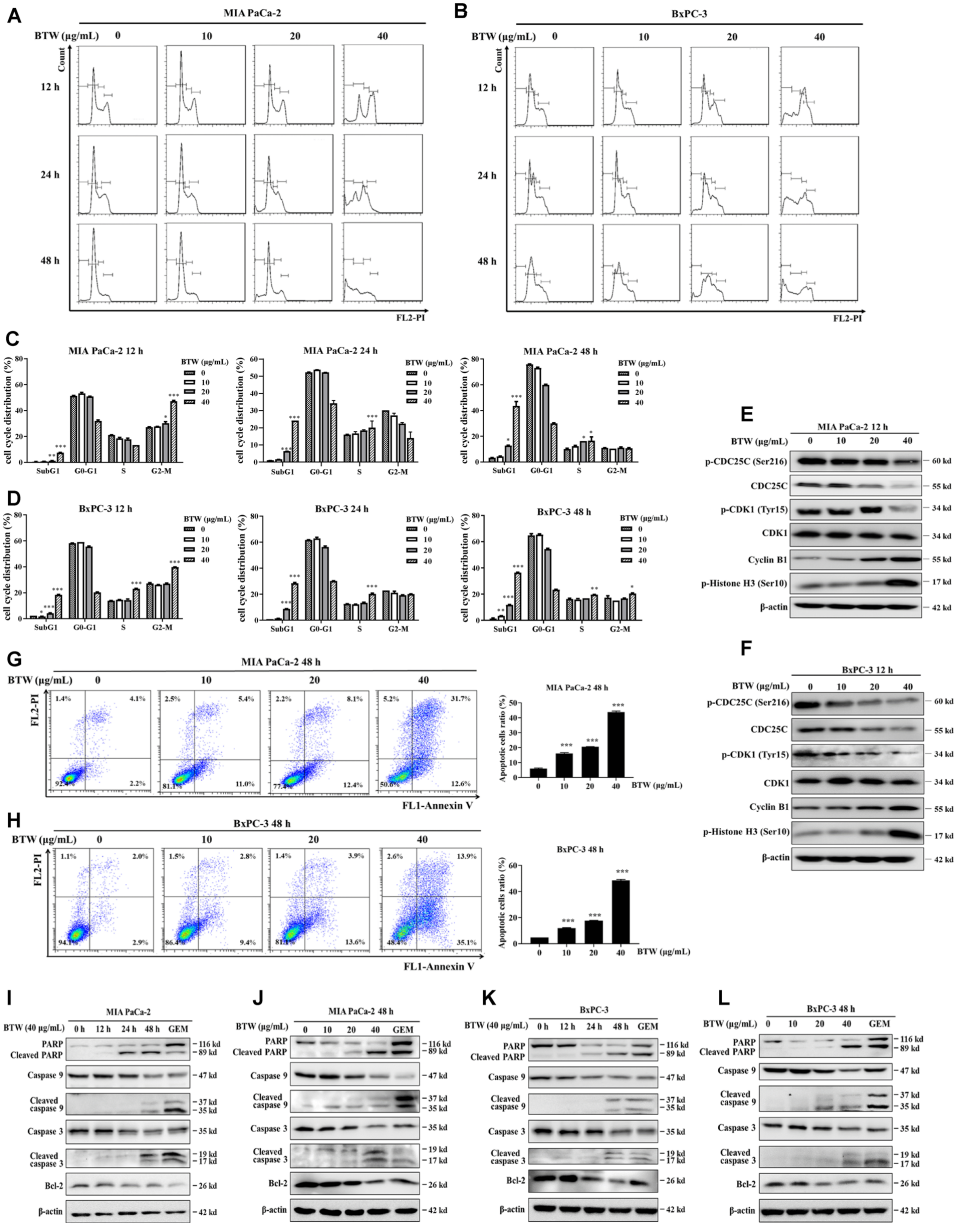
BTW induces G2/M cell cycle arrest and activates mitochondrial apoptosis in PAC cells. Representative images of cell cycle distribution of PAC cells (**A**, **B**) following BTW treatment at concentrations of 0, 10, 20 and 40 μg/mL for duration of 12, 24 and 48 h, respectively. Quantification data of cell cycle distribution of PAC cells (**C**, **D**). The expression of the indicated proteins in PAC cells (**E**, **F**) was analyzed using immunoblotting after treatment with 0–40 μg/mL of BTW for 12 h. The expression levels were normalized to β-actin. Double staining of Annexin V-FITC and PI was conducted to detect apoptotic cell death in PAC cells (**G**, **H**) following BTW treatment (0–40 μg/mL) for 48 h. Quantification analysis was shown on the right panel. The expression of apoptosis-related proteins was evaluated using immunoblotting in MIA PaCa-2 (**I**, **J**) and BxPC-3 (**K**, **L**) cells following BTW treatment. β-actin was used as an internal reference for protein expression normalization. 0.5 μM of GEM was chosen as the positive control. Results are presented as mean ± S.D. of triplicate independent experiments. *p < 0.05, **p < 0.01, ***p < 0.001 compared with the control

To examine whether the underlying anti-pancreatic mechanism of BTW was consistent with PD, we investigated the effects of BTW on inducing cell cycle arrest at G2/M phase and cellular apoptosis. In PAC cells following 12 h of treatment, BTW dose-dependently expanded the cell population in G2/M phase, with the proportion rising from 26.9% to 46.9% in MIA PaCa-2 cells and from 26.8% to 39.8% in BxPC-3 cells, respectively (Fig. 3A–D). Additionally, BTW reduced the protein levels of p-CDC25C (Ser216), CDC25C and p-CDK1 (Tyr15), while increased the protein expression of cyclin B1 and p-Histone H3 (Ser10) in the both cell lines (Fig. 3E, F). Moreover, BTW treatment for 24 h and 48 h remarkably accumulated PAC cells in the SubG1 phase (24.2% and 43.5% of MIA PaCa-2 cells, 28.5% and 36.4% of BxPC-3 cells) (Fig. 3A–D). Annexin V/PI staining assay detected that BTW dose-dependently caused a marked increase in apoptotic cells (Fig. 3G, H). The immunoblotting results consistently demonstrated that BTW dose- and time-dependently reduced Bcl-2 protein expression as well as activated PARP, caspase 3, and caspase 9, in both cell lines (Fig. 3I–L). Notably, treatment of either 40 μg/ml BTW or 200 μg/ml PD for 48 h started to stimulate the cleavage of PARP, caspase 3, and caspase 9 proteins, indicating that the antitumor action of 40 μg/ml BTW is comparable to 200 μg/ml PD. Collectively, the induction of cell cycle arrest at G2/M phase and caspase-dependent apoptosis in PAC cells by BTW shares similarities with the effects observed with PD, further supporting that BTW is the main active anti-pancreatic components in PD.

### β-peltatin, a small molecule extracted from BTW, shows strong anti-PAC actions by triggering G2/M phase arrest and apoptosis.

We then made an effort to identify the key chemical responsible for BTW action (a separate manuscript under review). Briefly, the aim compound showed same molecular ions with podophyllotoxin and picropodophyllotoxin, but the retention time and the major fragment ions were different from the two reference standards, suggesting the aim compound was an isomer of these two standards. LC-TOF–MS analysis of both commercial BTW extract and original BTW herb confirmed the presence of this isomer. Analysis using ^1^H-NMR and ^13^C-NMR revealed the aim compound as β-peltatin with molecular formula C_22_H_22_O_8_ and molecular weight 414.4.

**Fig. 4:**
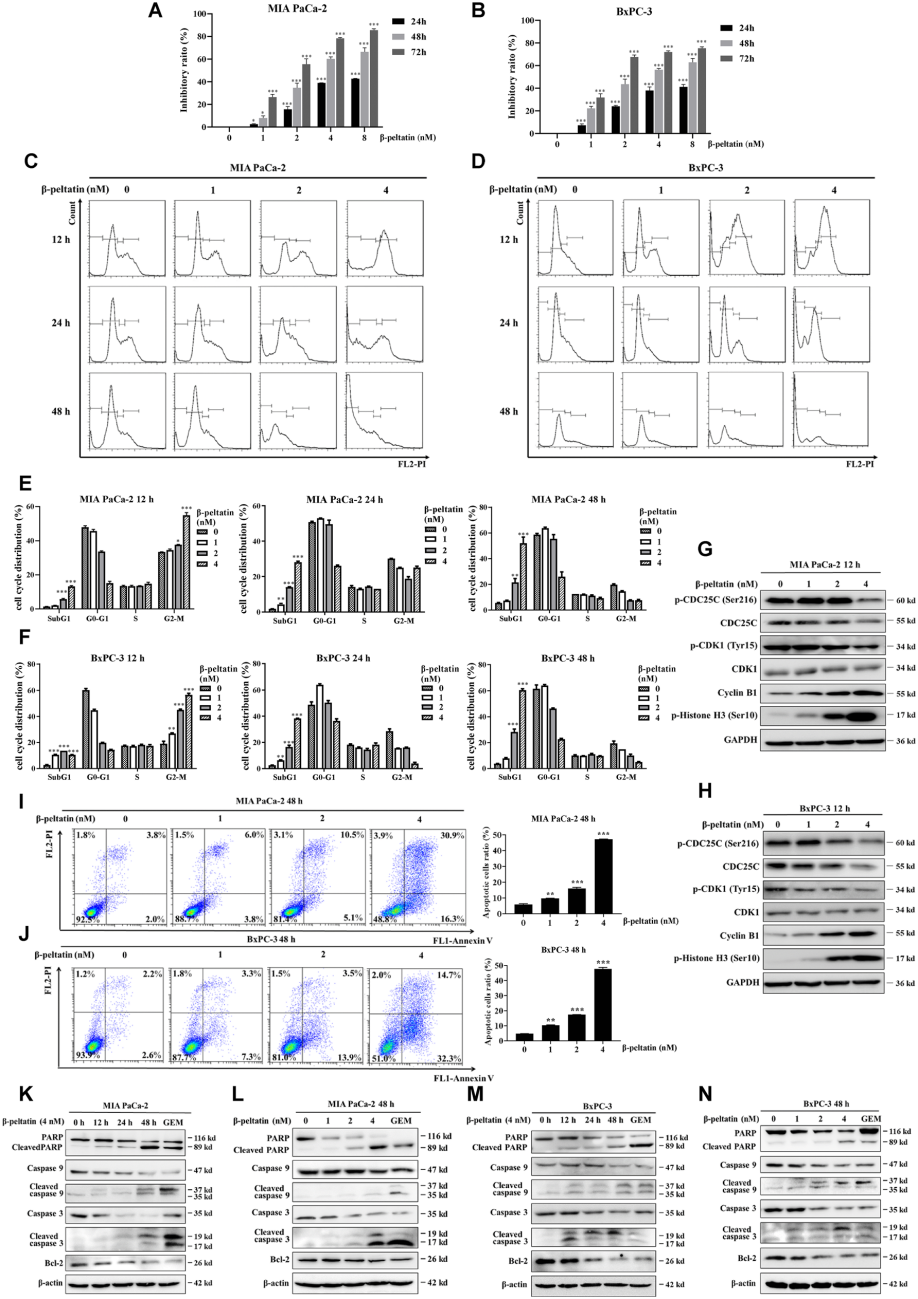
β-peltatin inhibits the growth of PAC cells by inducing G2/M cell cycle arrest and mitochondrial apoptosis. Cell viability of PAC cells (**A**, **B**) was examined following treatment with the indicated concentrations of β-peltatin for 24 h, 48 h and 72 h. Representative images of cell cycle distribution of PAC cells (**C**, **D**) following administration of 0–4 nM β-peltatin for 12–48 h, respectively. Quantification data of cell cycle distribution of PAC cells (**E**, **F**). The expression of G2/M phase-related proteins in PAC cells (**G**, **H**) were examined using immunoblotting following treatment with β-peltatin at concentrations of 0, 1, 2 and 4 nM for 12 h. The expression levels were normalized to GAPDH. Following 48 h of β-peltatin administration, the apoptotic PAC cells were assessed by Annexin V-FITC and PI-stained flow cytometry (**I**, **J**) and quantified (right panel). The expression levels of apoptosis-related proteins were evaluated by immunoblotting in MIA PaCa-2 (**K**, **L**) and BxPC-3 (**M**, **N**) cells with β-peltatin treatment at the indicated intervals and doses. The expression levels were normalized to β-actin. 0.5 μM of GEM was chosen as the positive control. Results are presented as mean ± S.D. of triplicate independent experiments. *p < 0.05, **p < 0.01, ***p < 0.001, compared with the control

Next, we exposed PAC cells to the indicated concentrations of β-peltatin for a period of 24 h, 48 h and 72 h (Fig. 4A, B). The IC_50_ value of β-peltatin (72 h treatment) was 2.09 nM ± 0.72 in MIA PaCa-2 and 1.49 nM ± 0.37 in BxPC-3 (Table 2), indicating its pronounced contribution to the cytotoxicity of BTW and PD. To verify β-peltatin is the main compound of BTW and PD that exerted cell cycle arrest at G2/M phase and apoptotic cell death induction on PAC cells, we evaluated the cell cycle distribution and the mechanism of cell death. The results in Fig. 4C–F showed that 12 h treatment of 2 nM β-peltatin significantly induced G2/M phase arrest. Annexin V/PI double staining results reflected that 4 nM β-peltatin induced about 40–50% PAC cells underwent apoptosis (Fig. 4I, J). Consistently, β-peltatin dose- and time-dependently modulated G2/M phase-related pathway (Fig. 4G, H) and mitochondria-mediated caspase-dependent pathway (Fig. 4K–N). The data collectively indicate the dominant role of β-peltatin in the anti-PAC activity of BTW and PD by promoting cell cycle arrest at G2/M phase and cellular apoptosis.

**Table 2:** IC_50_ values of β-peltatin and podophyllotoxin in PAC cells

Group	MIA PaCa-2		BxPC-3	
	48 h	72 h	48 h	72 h
β-peltatin (nM)	2.51 ± 0.54	2.09 ± 0.72	3.15 ± 0.67	1.49 ± 0.37
Podophyllotoxin (nM)	12.60 ± 0.54	11.33 ± 0.69	14.15 ± 0.23	13.71 ± 0.31

The values of IC_50_ were determined using GraphPad Prism 5 to analyze the results of the CCK-8 assay (Fig. 4A, B, Additional file 1: Fig. S3A, B). The IC_50_ values were obtained from the mean ± S.D. of triplicate experiments.

β-peltatin is the isomer of podophyllotoxin, an aryltetralin-type lignan isolated from species of *Podophyllum*, which exhibited potent anticancer actions but clinically obsoleted due to its serious toxicity [24]. We therefore compared the cytotoxicity of β-peltatin and podophyllotoxin on PAC cells by using the CCK8 assay (Fig. 4A, B, Additional file 1: Fig. S3A, B). The IC_50_ value of podophyllotoxin (11.33 nM ± 0.69 in MIA PaCa-2 cells, 13.71 nM ± 0.31 in BxPC-3 cells) were about 5- to 9-fold higher than that of β-peltatin (2.09 nM ± 0.72 in MIA PaCa-2 cells, 1.49 nM ± 0.37 in BxPC-3 cells) (Table 2). Next, we compared the acute toxicity of β-peltatin and podophyllotoxin in mice. Results showed that the overall survival rate of mice after a single administration of 60 mg/kg β-peltatin and 60 mg/kg podophyllotoxin was 90% and 60%, respectively (Additional file 1: Fig. S3C), despite no significant alterations in body weight of survived mice in either β-peltatin or podophyllotoxin-treated groups (Additional file 1: Fig. S3D). Therefore, these data support that comparing with podophyllotoxin, β-peltatin has stronger PAC cell proliferation inhibitory activity and lower acute toxicity in mice.

### β-peltatin suppresses tumor growth of BxPC-3 cells in vivo

**Fig. 5:**
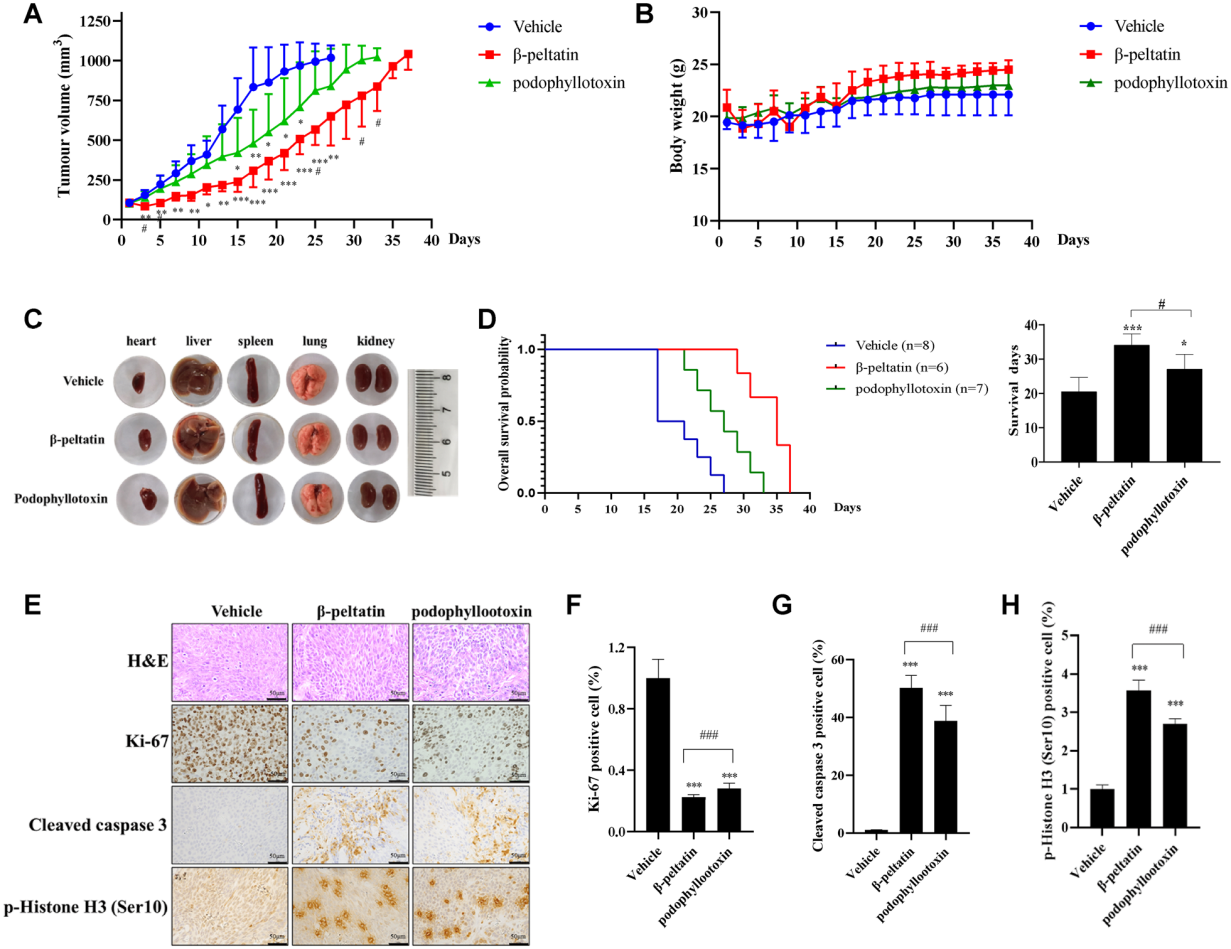
β-peltatin inhibits pancreatic tumor growth of BxPC-3 cells in vivo. BxPC-3 cells were administered *via* subcutaneous injection into the right flank of nude mice. Upon the tumor reaching a size of 100 mm^3^, mice were randomly divided into three different groups: vehicle, β-peltatin (15 mg/kg) and podophyllotoxin (15 mg/kg). Measurements of tumor volume (**A**) and mice body weight (**B**) were recorded every other day. *compared with vehicle group; ^#^statistical significance between β-peltatin and podophyllotoxin group. The mice were sacrificed when tumor volume reached 1000 mm^3^, the primary organs (**C**) were excised and photographed. Survival curves (**D**) and quantification data (right panel) for mice treated with vehicle, β-peltatin, and podophyllotoxin. Immunohistochemical analysis was conducted on paraffin-embedded tumor tissues using H&E staining, and antibodies against Ki-67, cleaved caspase 3 and p-Histone H3 (Ser10); **E**–**H** showed representative images (E, × 400) and quantification data (**F**–**H**). Results are presented as mean ± S.D. *p < 0.05, **p < 0.01, ***p < 0.001 compared with the control; ^#^*p* < 0.05, ^###^*p* < 0.001 compared with the indicated group

We used the subcutaneously-xenografted BxPC-3 cells model to examine in vivo anti-tumor effect of β-peltatin. Intraperitoneal administration of 15 mg/kg β-peltatin (once weekly) significantly suppressed tumor growth (Fig. 5A) without any notable alterations in the body weight of mice compared to control group (Fig. 5B) or gross anatomy of primary organs (Fig. 5C). Notably, when compared with its isomer podophyllotoxin, β-peltatin inhibited tumor growth more effectively. Moreover, β-peltatin prolonged the survival time of mice by 65.85% (34 days) and podophyllotoxin by 31.71% (27 days) compared to controls (21 days) (Fig. 5D). H&E and immunohistochemical staining of resected tumors from β-peltatin-treated mice displayed sparse cellularity and negative Ki-67 expression (Fig. 5E, F). Furthermore, β-peltatin treatment notably elevated the levels of cleaved caspase 3 and p-Histone H3 (Ser10) proteins in comparison to the vehicle control group (Fig. 5G, H), which is consistent with the in vitro results. These findings indicate that β-peltatin has the potential to suppress pancreatic tumor growth in vivo, reduces Ki-67 expression, and elevates the expression of cleaved caspase 3 and p-Histone H3 (Ser10) in tumor tissues.

## Discussion

In the present study, our data revealed that, of the 13 examined TCM herbal formulae, PD exerted the most potent cytotoxicity in two PAC cell lines. Further mechanistic studies revealed that the cytotoxicity of PD on PAC cells was attributable to induction of cell cycle arrest at G2/M phase and promotion of mitochondrial apoptosis. As PD comprises four herbs, we then validated that BTW was the key herbal constituent contributing to the anti-PAC action of PD. In a parallel study, analyzing the components of BTW allowed us to identify β-peltatin as the leading active component for its anti-cancer effect. Additionally, our findings confirmed that the anti-PAC action of PD, BTW and β-peltatin were consistently through down-regulation of proteins involved in G2/M phase regulation and apoptosis inhibition as well as up-regulation of cyclin B1 and p-Histone H3 (Ser10), resulting in cell cycle arrest and cellular apoptosis.

Despite the anticancer value of TCM formulae and its broad clinical application in China and neighboring countries, they are yet to be integrated into the mainstream treatment that prefer single active components [25]. The hurdles for TCM formulae to be integrated include their complex multi-constituent, actions on multi-target, and synergistic therapeutic actions of constituents in TCM formulae [26, 27]. Therefore, to expand the use of TCM formulae into mainstream neccesitates the identification of leading bioactive constituent. Several pieces of preclinical evidence have revealed that PD exhibits anticancer potential [4, 5]. However, the principal bioactive constituent responsible for its anti-cancer action is indistinct, due to its intricate multi-constituent nature. Through activity-based separation analysis, the present study provides evidence that BTW is the bioactive constituents for the anti-PAC effects of PD. Further separation of BTW extraction confirmed that a small molecule β-peltatin dominated the anti-PAC activities of PD. Determining single bioactive component, such as β-peltatin, is advantageous in boosting its progress from bench to bedside, and ultimately facilitating its integration in mainstay PAC treatment.

In this research, we compared the tumor suppressive effect and safety profiles of β-peltatin with podophyllotoxin. Podophyllotoxin, though having same molecular formula as of β-peltatin but is structurally different. Podophyllotoxin was isolated in 1880 and its structure only managed to be illustrated in 1930. Podophyllotoxin has a wide range of therapeutic properties, such as anticancer, antimicrobial, anti-inflammatory, lipid lowering, immunosuppressant, and antioxidative. However, podophyllotoxin exerts systemic toxicity, severe cytotoxicity on normal cells, and emergence of drug-resistance, which hampers its clinical application [24]. The present study discovered that compared to podophyllotoxin, β-peltatin possessed significantly higher tumor suppressive effect in vitro and in vivo. More importantly, the lower mortality rate, undetectable change in both body weight and anatomy of major organs in β-peltatin-treated mice has crucially implied the low toxicity of β-peltatin. These observations establish the safety profile of β-peltatin while attaining superior anti-PAC action than podophyllotoxin. The safety evidence of β-peltatin is critical in driving its development toward clinical application, as the poor safety profile of podophyllotoxin has bottlenecked its clinical application. Furthermore, the potent cytotoxicity of β-peltatin against PAC cells deserves further investigation. More innovative technologies, such as Omics, PROTAC and GST pull-down, could be utilized to explore more in-depth mechanisms and identify potential targets of PD in PAC cells.

This study discovered that the anti-pancreatic action of PD, BTW and β-peltatin are by triggering G2/M cell cycle arrest and cellular apoptosis. Immunoblotting results demonstrated downregulated the levels of G2/M phase-related proteins CDC25C, p-CDC25C (Ser216) and p-CDK1 (Tyr15), and upregulated the level of cyclin B1 protein. The activation of the CDK1/cyclin B1 complex propels the cell cycle from G2 to M phase. At the G2/M transition, the activation of CDK1 is catalyzed by CDC25C phosphatase [28]. Decrease of CDC25C by PD, BTW and β-peltatin disrupts the activation of CDK1 and impedes cell cycle progression, resulting in G2/M phase arrest. Although we noticed an increase in cyclin B at high dose of PD, BTW and β-peltatin, its significance is not clear, considering the CDK1 dephosphorylation by CDC25C phosphatase is key step in cyclin B-CDK1 complexes activation [29]. Moreover, it is known that p-Histone H3 (Ser10) appears at the peripheral nucleus in the late G2 phase and peaks in the middle of the M phase, which could sever as a biomarker of the M phase [22, 23]. The present study found an increased expression of p-Histone H3 (Ser10), indicating that PD, BTW and β-peltatin might arrest PAC cells mainly after the late G2 phase or at the M phase, which deserves further investigation.

This present study confirmed that PD, BTW and β-peltatin treatment induced G2/M cell cycle arrest at 12 h, while triggered apoptosis at 24 and 48 h. This indicated that PD, BTW and β-peltatin-induced apoptosis might be caused by G2/M arrest. However, more mechanistic studies are required to further specify whether PD, BTW and β-peltatin-induced apoptosis is caused by G2 or M phase. Additionally, PD, BTW and β-peltatin treatments decrease Bcl-2 protein expression, and activate caspase 3, caspase 9, and PARP. The decreased expression of the anti-apoptotic Bcl-2 protein implies that the regulation of mitochondrial apoptosis involves a coordinated modulation of both the anti-apoptotic and pro-apoptotic families of proteins [30].

## Conclusions

In conclusion, our findings explored the anti-pancreatic potential of PD by confirming its bioactive components and underlying mechanisms. BTW and β-peltatin dominate the anti-PAC action of PD by inducing G2/M cell cycle arrest and subsequently leading to apoptosis. Further mechanistic studies are required to facilitate suitable patient selection and optimize treatment response to β-peltatin in the future.

## Supplementary Information

**Additional file 1: Figure S1.** The composition and quality control of PD; **Table S1.** Composition of classical prescriptions; **Figure S2.** The cytotoxicity of 13 Chinese herbal formulae on pancreatic cancer cells; **Figure S3.** The cytotoxicity of podophyllotoxin and comparison of acute toxicity between β-peltatin and podo-phyllotoxin in mice.

## Data Availability

All data generated or analysed during this study are included in this published article (and its Additional files).

## Abbreviations

PAC: Pancreatic cancer
PD: Pulsatilla Decoction
HPLC: High performance liquid chromatography
CCK-8: Cell Counting Kit-8
TCM: Traditional Chinese Medicine
BTW: *Pulsatillae chinensis*, Bai Tou Weng
HL: *Coptidis chinensis*, Huang Lian
HB: *Phellodendri Chinensis Cortex,* Huang Bai
QP: *Fraxini Cortex*, Qin Pi
GEM: Gemcitabine HCl
PI: Propidium iodide
H&E: Hematoxylin and eosin

## References

[1] Siegel RL, Miller KD, Fuchs HE, Jemal A. Cancer statistics, 2022. CA Cancer J Clin. 2022;72(1):7–33. 35020204 10.3322/caac.21708

[2] Zhao Z, Liu W. Pancreatic cancer: a review of risk factors, diagnosis, and treatment. Technol Cancer Res Treat. 2020;19:1533033820962117. 33357065 10.1177/1533033820962117 PMC7768873

[3] McGuigan A, Kelly P, Turkington RC, Jones C, Coleman HG, McCain RS. Pancreatic cancer: a review of clinical diagnosis, epidemiology, treatment and outcomes. World J Gastroenterol. 2018;24(43):4846–61. 30487695 10.3748/wjg.v24.i43.4846 PMC6250924

[4] Jie Y, Yang X, Chen W. Pulsatilla decoction combined with 5-fluorouracil triggers immunogenic cell death in colorectal cancer cells. Cancer Biother Radiopharm. 2022;37(10):945–54. 34042519 10.1089/cbr.2020.4369

[5] Liu H, Hu Y, Qi B, Yan C, Wang L, Zhang Y, et al. Network pharmacology and molecular docking to elucidate the mechanism of pulsatilla decoction in the treatment of colon cancer. Front Pharmacol. 2022;13: 940508. 36003525 10.3389/fphar.2022.940508 PMC9393233

[6] Niu C, Hu XL, Yuan ZW, Xiao Y, Ji P, Wei YM, et al. Pulsatilla decoction improves dss-induced colitis via modulation of fecal-bacteria-related short-chain fatty acids and intestinal barrier integrity. J Ethnopharmacol. 2023;300: 115741. 36162543 10.1016/j.jep.2022.115741

[7] Yu Y, Chen J, Zhang X, Wang Y, Wang S, Zhao L, et al. Identification of anti-inflammatory compounds from zhongjing formulae by knowledge mining and high-content screening in a zebrafish model of inflammatory bowel diseases. Chin Med. 2021;16(1):42. 34059101 10.1186/s13020-021-00452-z PMC8166029

[8] Hua YL, Ma Q, Zhang XS, Jia YQ, Peng XT, Yao WL, et al. Pulsatilla decoction can treat the dampness-heat diarrhea rat model by regulating glycerinphospholipid metabolism based lipidomics approach. Front Pharmacol. 2020;11:197. 32194420 10.3389/fphar.2020.00197 PMC7064006

[9] Liu X, He S, Li Q, Mu X, Hu G, Dong H. Comparison of the gut microbiota between Pulsatilla decoction and levofloxacin hydrochloride therapy on *Escherichia**coli* infection. Front Cell Infect Microbiol. 2020;10:319. 32714880 10.3389/fcimb.2020.00319 PMC7344306

[10] Hu K, Zhang H, Shi G, Wang B, Wu D, Shao J, et al. Effects of n-butanol extract of pulsatilla decoction on the nlrp3 inflammasome in macrophages infected with *Candida**albicans*. J Ethnopharmacol. 2023;304: 116041. 36539072 10.1016/j.jep.2022.116041

[11] Deng LR, Han Q, Zou M, Chen FJ, Huang CY, Zhong YM, et al. Identification of potential immunomodulators from Pulsatilla decoction that act on therapeutic targets for ulcerative colitis based on pharmacological activity, absorbed ingredients, and in-silico molecular docking. Chin Med. 2022;17(1):132. 36434688 10.1186/s13020-022-00684-7 PMC9701001

[12] Li YH, Zou M, Han Q, Deng LR, Weinshilboum RM. Therapeutic potential of triterpenoid saponin anemoside b4 from *Pulsatilla**chinensis*. Pharmacol Res. 2020;160: 105079. 32679180 10.1016/j.phrs.2020.105079

[13] Liu P, Liu Y, Chen L, Fan Z, Luo Y, Cui Y. Anemoside a3 inhibits macrophage m2-like polarization to prevent triple-negative breast cancer metastasis. Molecules. 2023;28(4):1611. 36838599 10.3390/molecules28041611 PMC9967222

[14] Yin L, Fan Z, Liu P, Chen L, Guan Z, Liu Y, et al. Anemoside a3 activates tlr4-dependent m1-phenotype macrophage polarization to represses breast tumor growth and angiogenesis. Toxicol Appl Pharmacol. 2021;432: 115755. 34673087 10.1016/j.taap.2021.115755

[15] Zhu C, Li K, Peng XX, Yao TJ, Wang ZY, Hu P, et al. Berberine a traditional chinese drug repurposing: its actions in inflammation-associated ulcerative colitis and cancer therapy. Front Immunol. 2022;13:1083788. 36561763 10.3389/fimmu.2022.1083788 PMC9763584

[16] Achi IT, Sarbadhikary P, George BP, Abrahamse H. Multi-target potential of berberine as an antineoplastic and antimetastatic agent: a special focus on lung cancer treatment. Cells. 2022;11(21):3433. 36359829 10.3390/cells11213433 PMC9655513

[17] Ma Z, Sun Y, Peng W. Fraxetin down-regulates polo-like kinase 4 (PLK4) to inhibit proliferation, migration and invasion of prostate cancer cells through the phosphatidylinositol 3-kinase (PI3K)/protein kinase b (AKT) pathway. Bioengineered. 2022;13(4):9345–56. 35387563 10.1080/21655979.2022.2054195 PMC9161838

[18] Yang L, Wu H, Qiu W, Guo L, Du X, Yu Q, et al. Pulsatilla decoction inhibits *Candida**albicans* proliferation and adhesion in a mouse model of vulvovaginal candidiasis via the dectin-1 signaling pathway. J Ethnopharmacol. 2018;223:51–62. 29775695 10.1016/j.jep.2018.05.018

[19] Jiang X, Li Y, Feng JL, Nik Nabil WN, Wu R, Lu Y, et al. Safranal prevents prostate cancer recurrence by blocking the re-activation of quiescent cancer cells via downregulation of s-phase kinase-associated protein 2. Front Cell Dev Biol. 2020;8: 598620. 33392189 10.3389/fcell.2020.598620 PMC7772204

[20] Feng J, Xi Z, Jiang X, Li Y, Nik Nabil WN, Liu M, et al. Saikosaponin a enhances docetaxel efficacy by selectively inducing death of dormant prostate cancer cells through excessive autophagy. Cancer Lett. 2023;554: 216011. 36442771 10.1016/j.canlet.2022.216011

[21] Zhang SB, Hong M, Sun XY, Huang D, He DH, Chen YF, et al. Silybin has therapeutic efficacy against non-small cell lung cancer through targeting of skp2. Acta Mater Med. 2022;1(3):302–13.

[22] Lindqvist A, Rodriguez-Bravo V, Medema RH. The decision to enter mitosis: feedback and redundancy in the mitotic entry network. J Cell Biol. 2009;185(2):193–202. 19364923 10.1083/jcb.200812045 PMC2700378

[23] Prigent C, Dimitrov S. Phosphorylation of serine 10 in histone h3, what for? J Cell Sci. 2003;116(18):3677–85. 12917355 10.1242/jcs.00735

[24] Shah Z, Gohar UF, Jamshed I, Mushtaq A, Mukhtar H, Zia-Ui-Haq M, et al. Podophyllotoxin: history, recent advances and future prospects. Biomolecules. 2021;11(4):603. 33921719 10.3390/biom11040603 PMC8073934

[25] Liang Z, Lai Y, Li M, Shi J, Lei CL, Hu H, et al. Applying regulatory science in traditional chinese medicines for improving public safety and facilitating innovation in China: a scoping review and regulatory implications. Chin Med. 2021;16(1):23. 33593397 10.1186/s13020-021-00433-2 PMC7884970

[26] Yao CL, Zhang JQ, Li JY, Wei WL, Wu SF, Guo DA. Traditional chinese medicine (TCM) as a source of new anticancer drugs. Nat Prod Rep. 2021;38(9):1618–33. 33511969 10.1039/d0np00057d

[27] Lin AX, Chan G, Hu Y, Ouyang D, Ung CO, Shi L, et al. Internationalization of traditional Chinese medicine: current international market, internationalization challenges and prospective suggestions. Chin Med. 2018;13(1):9. 29449877 10.1186/s13020-018-0167-z PMC5807832

[28] Forester CM, Maddox J, Louis JV, Goris J, Virshup DM. Control of mitotic exit by pp2a regulation of cdc25c and cdk1. Proc Natl Acad Sci USA. 2007;104(50):19867–72. 18056802 10.1073/pnas.0709879104 PMC2148389

[29] Zhou F, Aipire A, Xia L, Halike X, Yuan P, Sulayman M, et al. *Marchantia**polymorpha* L. ethanol extract induces apoptosis in hepatocellular carcinoma cells via intrinsic- and endoplasmic reticulum stress-associated pathways. Chin Med. 2021;16(1):94. 34583719 10.1186/s13020-021-00504-4 PMC8477563

[30] Chen X, Zeh HJ, Kang R, Kroemer G, Tang D. Cell death in pancreatic cancer: from pathogenesis to therapy. Nat Rev Gastroenterol Hepatol. 2021;18(11):804–23. 34331036 10.1038/s41575-021-00486-6

