# Correction: Pulsatilla Decoction and its bioactive component β-peltatin induce G2/M cell cycle arrest and apoptosis in pancreatic cancer

**DOI:** 10.1186/s13020-025-01207-w

**Published:** 2025-11-20

**Authors:** Rong Wu, Zhichao Xi, Mengfan Liu, Hangui Ren, Rongchen Dai, Xue Jiang, Wan Najbah Nik Nabil, Yalin Wang, Jiling Feng, Qiong Chai, Qihan Dong, Hongxi Xu

**Affiliations:** 1 School of Pharmacy, Shanghai University of Traditional Chinese Medicine, Shanghai, 201203 China; 2 Engineering Research Center of Shanghai Colleges for TCM New Drug Discovery, Shanghai, 201203 China; 3 Pharmaceutical Services Program, Ministry of Health, Petaling Jaya, Selangor, 46200 Malaysia; 4 Chinese Medicine Anti-Cancer Evaluation Program, Greg Brown Laboratory, Central Clinical School and Charles Perkins Centre, Faculty of Medicine and Health, The University of Sydney, Sydney, NSW 2006 Australia; 5 Department of Endocrinology, Royal Prince Alfred Hospital, Sydney, NSW 2050 Australia

**Correction** **:** **Chinese Medicine (2023) 18:61** 10.1186/s13020-023-00774-0

Following publication of the original article [1], the authors found that the image for BxPC-3 treated with PD at 100 μg/mL was inadvertently duplicated from the 50 μg/mL image during figure processing, resulting in an unintentional overlap in Fig. 1J. In Fig. 4I and J, the images of the control sample have been replaced to avoid any potential misinterpretation.

The incorrect Fig. 1 is:

**Fig. 1:**
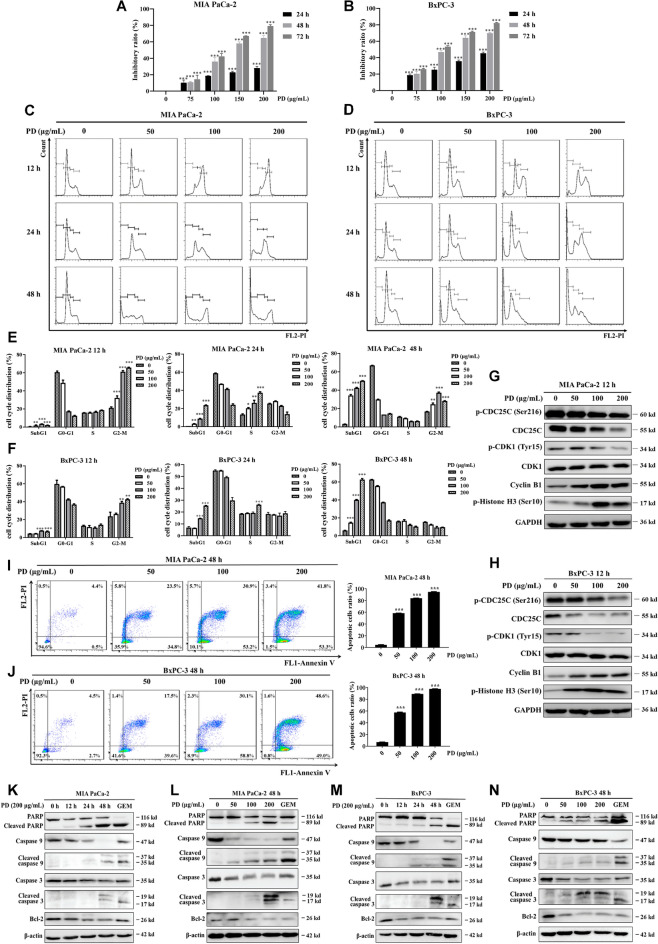
PD induces cell cycle arrest at G2/M phase and mitochondrial apoptosis in PAC cells. The viability of PAC cells (**A**, **B**) after PD treatment at concentrations of 0, 75, 100, 150 and 200 μg/mL was determined using CCK-8 for 24 h, 48 h and 72 h, respectively. Representative images of cell cycle distribution of PAC cells (**C**, **D**) after treated with PD (0–200 μg/mL) for 12–48 h, respectively. Quantification data of cell cycle distribution for PAC cells (**E**, **F**) are presented. The levels of G2/M phase-related regulatory proteins (p-CDC25C (Ser216), CDC25C, Cyclin B1, p-CDK1(Tyr15), CDK1 and p-Histone H3 (Ser10)) in PAC cells (G, H) were analyzed using immunoblotting following exposure to PD at concentrations of 0, 50, 100 and 200 μg/mL for 12 h. The expression levels were normalized to GAPDH. Double staining with Annexin V-FITC and PI was conducted to evaluate apoptosis in PAC cells (**I**, **J**) following PD treatment for 48 h (0–200 μg/mL) and then quantified (right panel). Apoptosis-associated protein expression was determined using immunoblotting in MIA PaCa-2 (**K**, **L**) and BxPC-3 (**M**, **N**) following PD treatment at concentrations of 0, 50, 100 and 200 μg/mL for duration of 12, 24 and 48 h. The expression levels were normalized to β-actin. 0.5 μM of GEM was chosen as the positive control. Results are presented as mean ± S.D. of triplicate independent experiments. *p < 0.05, **p < 0.01, ***p < 0.001 compared with the control

The correct Fig. 1 is:

**Fig. 1:**
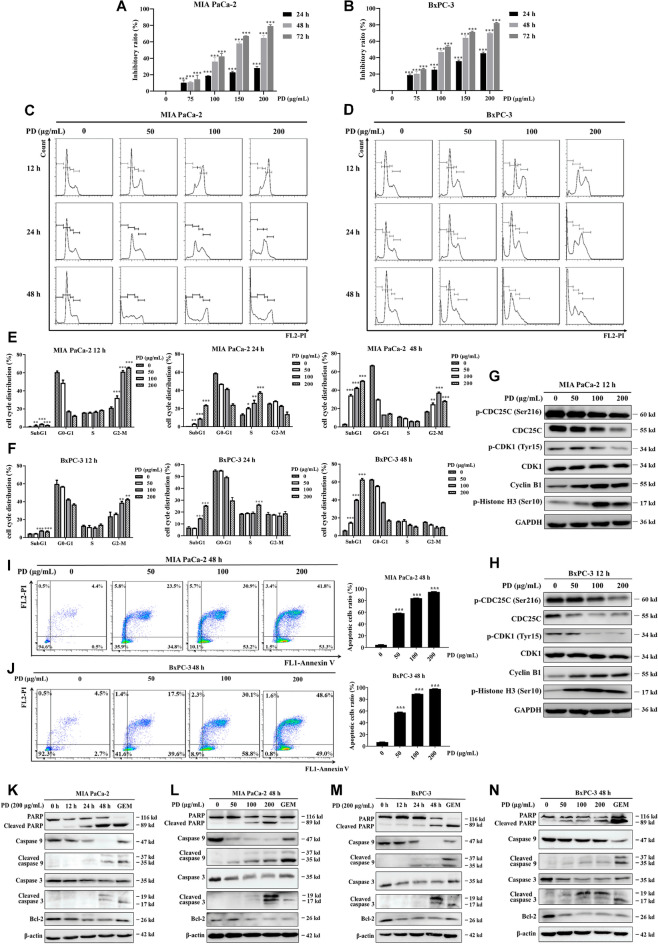
PD induces cell cycle arrest at G2/M phase and mitochondrial apoptosis in PAC cells. The viability of PAC cells (**A**, **B**) after PD treatment at concentrations of 0, 75, 100, 150 and 200 μg/mL was determined using CCK-8 for 24 h, 48 h and 72 h, respectively. Representative images of cell cycle distribution of PAC cells (**C**, **D**) after treated with PD (0–200 μg/mL) for 12–48 h, respectively. Quantification data of cell cycle distribution for PAC cells (**E**, **F**) are presented. The levels of G2/M phase-related regulatory proteins (p-CDC25C (Ser216), CDC25C, Cyclin B1, p-CDK1(Tyr15), CDK1 and p-Histone H3 (Ser10)) in PAC cells (**G**, **H**) were analyzed using immunoblotting following exposure to PD at concentrations of 0, 50, 100 and 200 μg/mL for 12 h. The expression levels were normalized to GAPDH. Double staining with Annexin V-FITC and PI was conducted to evaluate apoptosis in PAC cells (**I**, **J**) following PD treatment for 48 h (0–200 μg/mL) and then quantified (right panel). Apoptosis-associated protein expression was determined using immunoblotting in MIA PaCa-2 (**K**, **L**) and BxPC-3 (**M**, **N**) following PD treatment at concentrations of 0, 50, 100 and 200 μg/mL for duration of 12, 24 and 48 h. The expression levels were normalized to β-actin. 0.5 μM of GEM was chosen as the positive control. Results are presented as mean ± S.D. of triplicate independent experiments. *p < 0.05, **p < 0.01, ***p < 0.001 compared with the control

The incorrect Fig. 4 is:

**Fig. 4:**
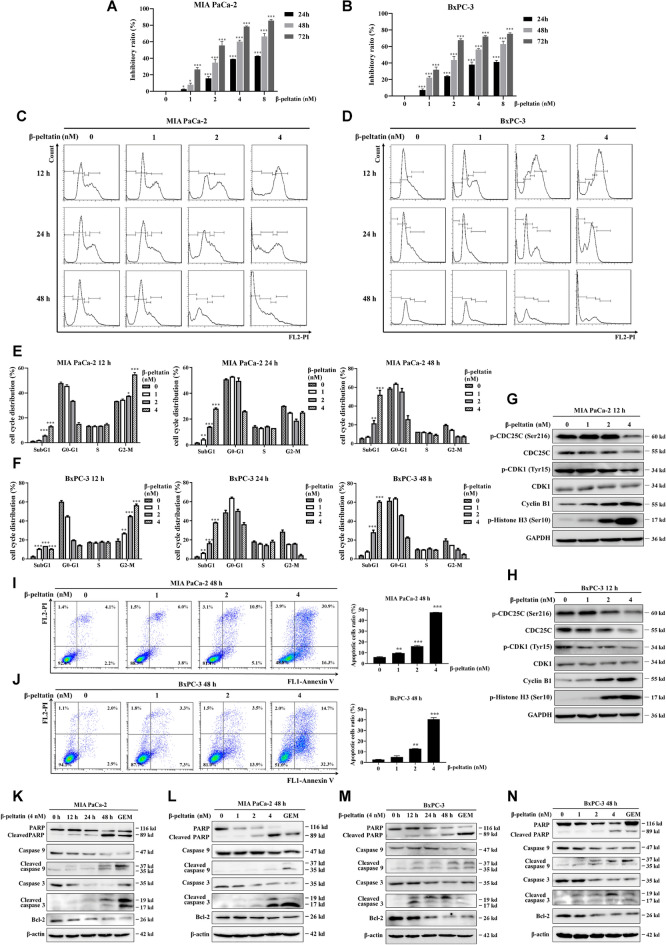
β-peltatin inhibits the growth of PAC cells by inducing G2/M cell cycle arrest and mitochondrial apoptosis. Cell viability of PAC cells (**A**, **B**) was examined following treatment with the indicated concentrations of β-peltatin for 24 h, 48 h and 72 h. Representative images of cell cycle distribution of PAC cells (**C**, **D**) following administration of 0–4 nM β-peltatin for 12–48 h, respectively. Quantification data of cell cycle distribution of PAC cells (**E**, **F**). The expression of G2/M phase-related proteins in PAC cells (**G**, **H**) were examined using immunoblotting following treatment with β-peltatin at concentrations of 0, 1, 2 and 4 nM for 12 h. The expression levels were normalized to GAPDH. Following 48 h of β-peltatin administration, the apoptotic PAC cells were assessed by Annexin V-FITC and PI-stained flow cytometry (**I**, **J**) and quantified (right panel). The expression levels of apoptosis-related proteins were evaluated by immunoblotting in MIA PaCa-2 (**K**, **L**) and BxPC-3 (**M**, **N**) cells with β-peltatin treatment at the indicated intervals and doses. The expression levels were normalized to β-actin. 0.5 μM of GEM was chosen as the positive control. Results are presented as mean ± S.D. of triplicate independent experiments. *p < 0.05, **p < 0.01, ***p < 0.001, compared with the control

The correct Fig. 4 is:

**Fig. 4:**
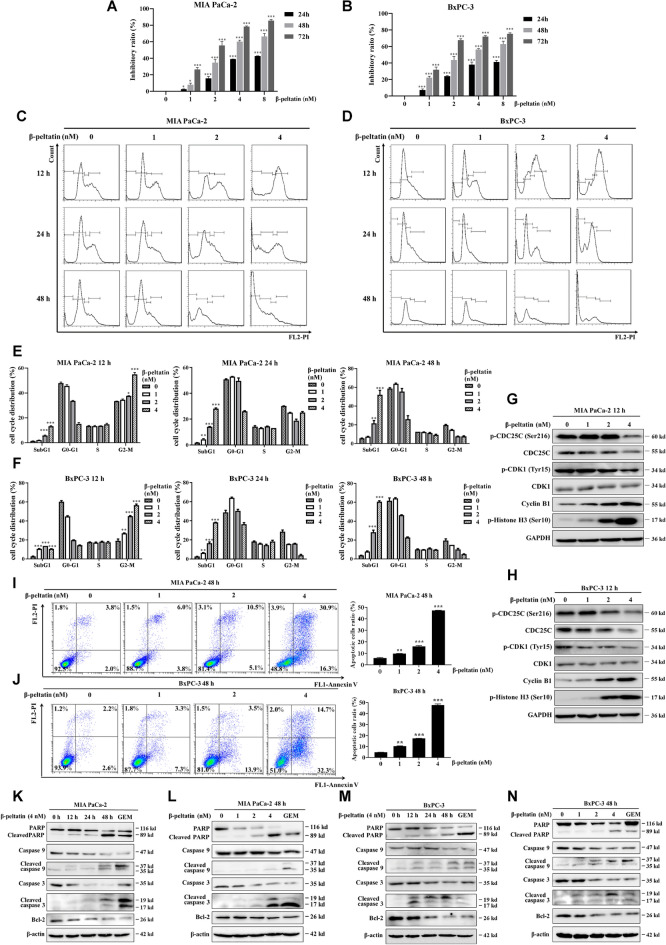
β-peltatin inhibits the growth of PAC cells by inducing G2/M cell cycle arrest and mitochondrial apoptosis. Cell viability of PAC cells (**A**, **B**) was examined following treatment with the indicated concentrations of β-peltatin for 24 h, 48 h and 72 h. Representative images of cell cycle distribution of PAC cells (**C**, **D**) following administration of 0–4 nM β-peltatin for 12–48 h, respectively. Quantification data of cell cycle distribution of PAC cells (**E**, **F**). The expression of G2/M phase-related proteins in PAC cells (**G**, **H**) were examined using immunoblotting following treatment with β-peltatin at concentrations of 0, 1, 2 and 4 nM for 12 h. The expression levels were normalized to GAPDH. Following 48 h of β-peltatin administration, the apoptotic PAC cells were assessed by Annexin V-FITC and PI-stained flow cytometry (**I**, **J**) and quantified (right panel). The expression levels of apoptosis-related proteins were evaluated by immunoblotting in MIA PaCa-2 (**K**, **L**) and BxPC-3 (**M**, **N**) cells with β-peltatin treatment at the indicated intervals and doses. The expression levels were normalized to β-actin. 0.5 μM of GEM was chosen as the positive control. Results are presented as mean ± S.D. of triplicate independent experiments. *p < 0.05, **p < 0.01, ***p < 0.001, compared with the control

The original article [1] has been corrected.
