# Supplementary material for: Pulsatilla Decoction and its bioactive component β-peltatin induce G2/M cell cycle arrest and apoptosis in pancreatic cancer
Source: Chin Med. 2023 May 28;18:61. doi: 10.1186/s13020-023-00774-0 (PMC10225094; doi:10.1186/s13020-023-00774-0)
Supplement: Supplementary file 1 — Additional file 1: Figure S1. The composition and quality control of PD; Table S1. Composition of classical prescriptions; Figure S2. The cytotoxicity of 13 Chinese herbal formulae on pancreatic cancer cells; Figure S3. The cytotoxicity of podophyllotoxin and comparison of acute toxicity between β-peltatin and podo-phyllotoxin in mice. [file 13020_2023_774_MOESM1_ESM.docx]

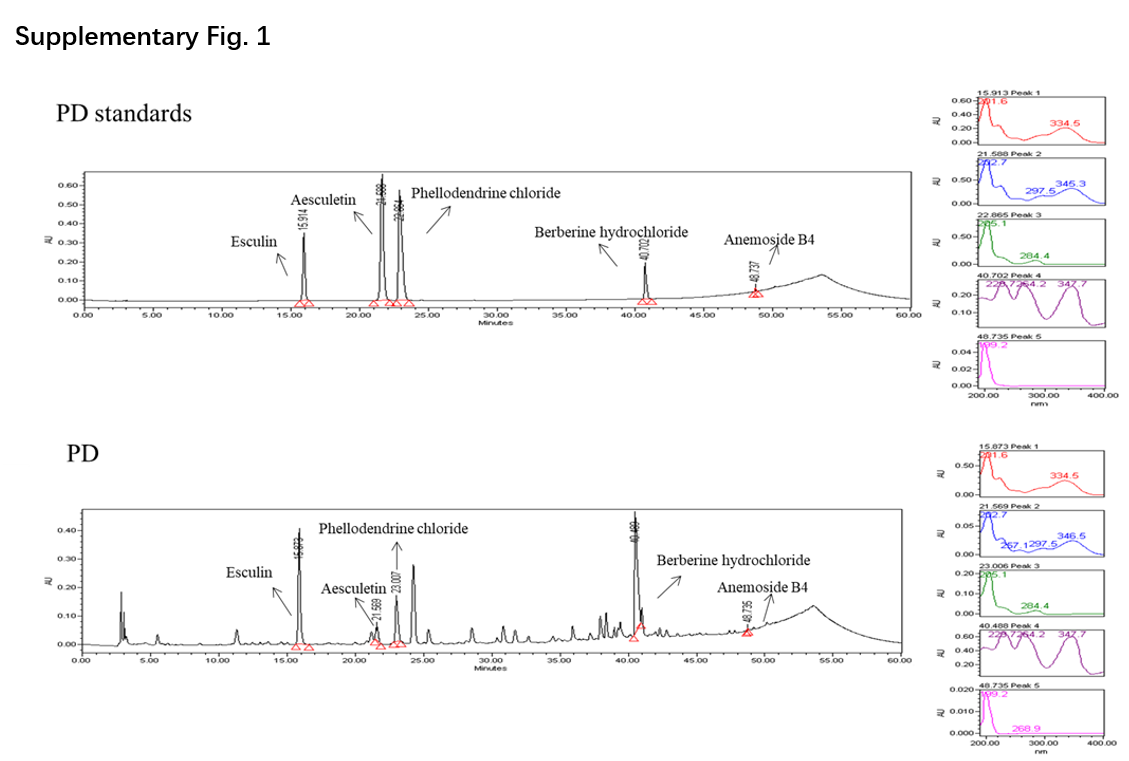


**Fig. S1. The composition and quality control of PD.** HPLC analysis showed that PD contained the five pharmacopoeia reference substances (esculin, aesculetin, phellodendrine hydrochloride, berberine hydrochloride and anemoside B4), as evidenced by comparing the peaks’ retention times and ultraviolet absorption between PD solution and the reference substances.

**Table S1. Composition of classical prescriptions**

| group | herbal formula name |
| --- | --- |
| A | Pulsatilla Decoction |
| B | Sini Decoction |
| C | Yinchenhao Tang Decoction |
| D | Zhi-Zi-Hou-Pu Decoction |
| E | Guizhi-Fuling Capsule |
| F | Xiaojianzhong Decoction |
| G | Lizhong Pill |
| H | Wuzhuyu Decoction |
| I | Wuling Capsule |
| J | Zhenwu Decoction |
| K | Linggui Zhugan Decoction |
| L | Sini Powder |
| M | Xiaochaihu Decoction |

Thirteen classical Chinese herbal formulae (A-M) from Chinese medicine book “Shang Han Treatise”.


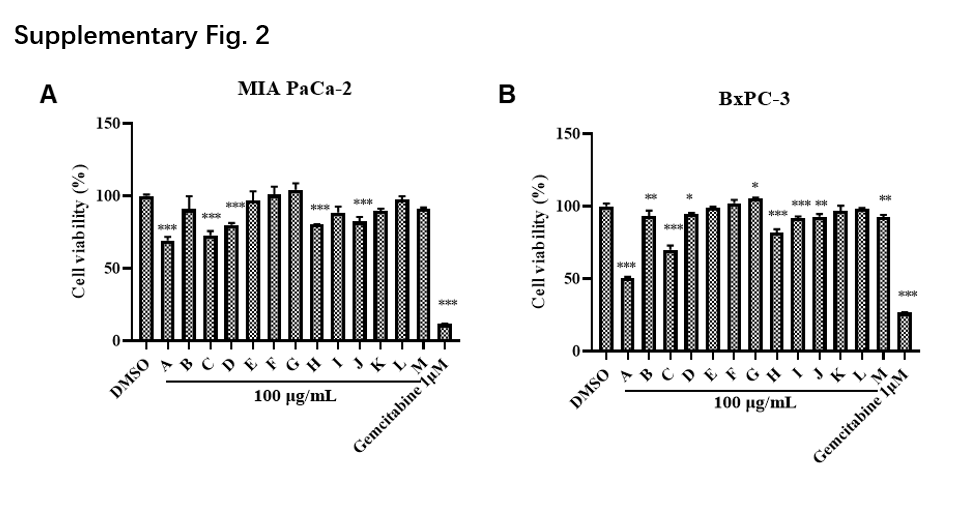


**Fig. S2. The cytotoxicity of 13 Chinese herbal formulae on pancreatic cancer cells.** CCK-8 assay was performed to measure the cell viability of MIA PaCa-2 (A) and BxPC-3 (B) after treated with 100 μg/mL of the indicated Chinese herbal formulae for 72 h. 1 μM of Gemcitabine (GEM) was chosen as the positive control. Data are presented as mean ± S.D. (n = 3). **p* < 0.05, ***p* < 0.01, ****p* < 0.001 compared with the control.


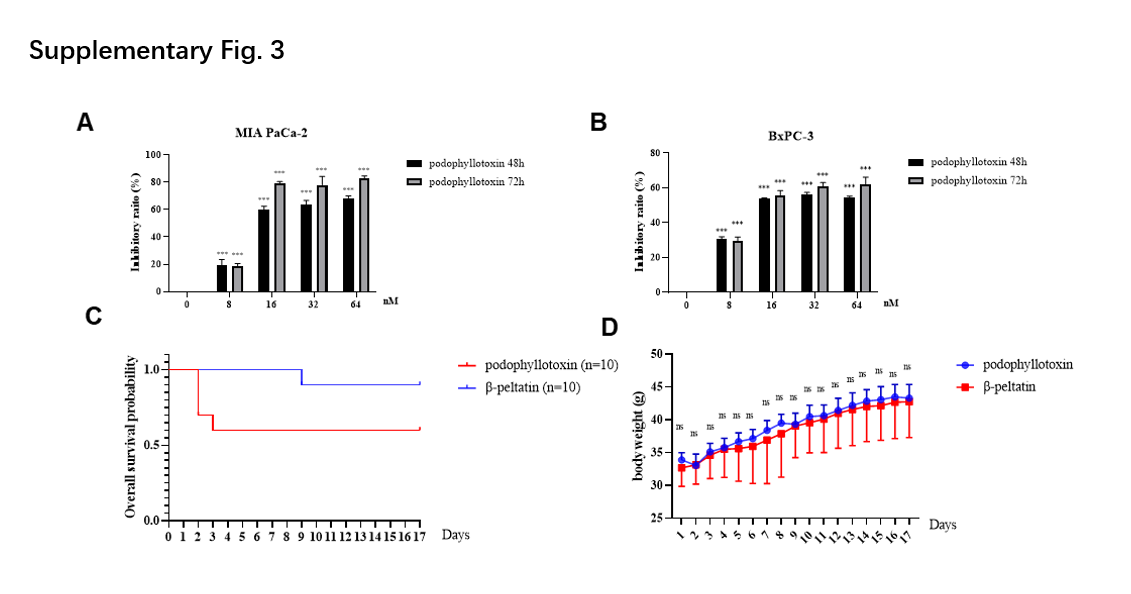


**Fig. S3: The cytotoxicity of podophyllotoxin and comparison of acute toxicity between β-peltatin and podophyllotoxin in mice.** CCK-8 assay was performed to measure the viability of MIA PaCa-2 (A) and BxPC-3 (B) cells after treated with podophyllotoxin at the indicated concentrations (0-64 nM) for 48 h and 72 h, respectively. Twenty BALB/c mice were randomly divided into two groups after fasted for 12-16 h. Mice were subcutaneously injected with 60 mg/kg of β-peltatin and podophyllotoxin and survival curves (C) were then plotted. (D) Mice body weight were recorded daily for 17 days. Data are presented as mean ± S.D. (n=3). ****p* < 0.001 compared with the control; ns, not significant.
